# Biomimetic HA-Reinforced PCL/PLA Fibrous Structures via Solution Blow Spinning: Cocoon-Inspired Design for Bone Regeneration

**DOI:** 10.1021/acsomega.5c06418

**Published:** 2025-11-21

**Authors:** Carolina L. Almeida, Lucas R. F. Figueiredo, José D. D. Melo, Eliton S. Medeiros

**Affiliations:** † Materials and Biosystems Laboratory (LAMAB), Department of Materials Engineering (DEMAT), 28123 Federal University of Paraiba (UFPB), João Pessoa, Paraíba 58051-900, Brazil; ‡ Post-Graduation Program in Materials Science and Engineering (PPCEM), Federal University of Paraíba (UFPB), João Pessoa, Paraíba 58051-900, Brazil; § Post-Graduation Program in Materials Science and Engineering (PPGCEM), Center of Exact and Earth Sciences (CCET), Federal University of Rio Grande do Norte (UFRN), Natal, Rio Grande do Norte 59078-970, Brazil

## Abstract

Bioinspired materials have been studied for applications in various fields, including aerospace and tissue engineering. In this study, biomimetic fibrous structures, inspired by the cocoons of the *Rothschildia* sp. moths, were produced by solution blow spinning (SBS) using biodegradable polymers such as polycaprolactone (PCL) and poly­(lactic acid) (PLA), selected for their distinct melting temperatures, enabling the fabrication of hot-pressed fiber-reinforced composites, with the addition of hydroxyapatite (HA) microparticles, due to its osteoconductive capacity. Cocoons were analyzed using scanning electron microscopy (SEM), tensile tests, Fourier-transform infrared spectroscopy (FTIR), and thermogravimetry (TG) and differential scanning calorimetry (DSC). The results showed that cocoons consist of fibroin dispersed in a sericin matrix, interspersed with HA particles, forming a fibrous multilayered structure. To replicate this structure, fibrous layers were produced using a multinozzle SBS system that spun PLA (fibrous phase) and PCL (matrix). A hot-press process at varying temperatures and spun layers was applied to melt PCL while preserving the fibrous morphology of PLA. Characterization of the bioinspired structures followed the same techniques used for the cocoons, revealing similar morphology with PLA fibers in a PCL-HA matrix. Mechanical strength varied, with the six-layer fibrous membrane, pressed at 90 °C for 30 min, exhibiting the highest strength. Overall, this research demonstrates the potential of developing bioinspired nanocomposites with customized properties based on the structure of moth cocoons.

## Graphical abstract

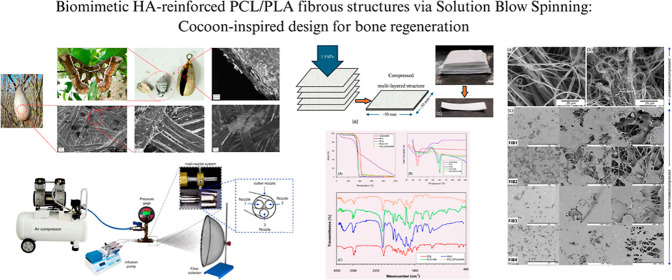

## 1 Introduction

Nature has produced complex and efficient materials such as spider’s webs and the adhesive ability of gecko’s feet, which serve as sources of inspiration for the development of new materials and structures.^1–3^ Among them, there is a species of moth from the *Saturniidae* family, genus *Rothschildia speculifer*, found in the Northeastern region of Brazil whose cocoons exhibit a morphology of ribbon-like fibers joined together by a biding matrix. These cocoons not only provide protection against falls and predator attacks but also supply hydroxyapatite (HA) for moth’s further development stages when undergoing metamorphosis from egg, larva (caterpillar), pupa (cocoon) to adult.^4,5^ The ribbon-like fiber structure observed in the cocoons serves as a natural composite model inspiring the development of high-performance materials. Bioinspired or biomimetic materials have been extensively studied across multiple fields to replicate properties refined by nature over the years. The goal of bioinspired composites (biocomposites) is to combine conventional materials with fibrous or particles reinforcements, achieving enhanced strength, lightweight, and multifunctionality.^6,7^

Fiber-reinforced composites can be fabricated using techniques such as solution blow spinning (SBS) and electrospinning (ES) to mimic cocoon architecture, with the specific aim of developing materials for bone tissue regeneration. This application potential stems from the osteoconductive capacity of hydroxyapatite and the ability to create fibrous polymer scaffolds that replicate the structural organization observed in natural cocoons.^8,9^ The SBS process uses a coaxial nozzle design, whereby, a polymer solution is pumped through the core and pressurized air fed through the outer annulus, making use of Bernoulli’s principle in which the change in air pressure is converted into kinetic energy of the polymer jet, accelerating and enabling fiber formation. As the high-pressure gas stream exits the outer nozzle, the pressure drops thereby increasing the kinetic energy of the stream resulting in an increase in the velocity of the gas that promotes a pressure drop at the center of the inner nozzle and draws the polymer solution into a cone shape, known as a solution cone similar in shape to Taylor cone formed during electrospinning.^8,10,11^ The high velocity gas induces shearing and drag forces at the gas/polymer solution interface. When the surface tension is overcome by these forces at the tip of the cone, fine threads of polymer solution are driven into the air stream, across the working distance, while being stretched to form fibers upon solvent evaporation.^12,13^ These fibers are then gathered together on a collector.^13,14^ The smaller the size of these fibers, the larger their surface area, making them a strategic material for certain applications and structures which can be further enhanced by adding reinforcements.^13,15,16^ Coupling this technique with mineral additives (e.g., hydroxyapatite) can replicate the efficiency of natural composites, paving the way for sustainable, high-performance materials.^10,15,17^

The incorporation of hydroxyapatite (HA) microparticles into these composites enables the development of a biomimetic material that closely replicates the natural structure of cocoons. The integration of HA with polymers yields a biodegradable material featuring a fiber morphology that promotes cell proliferation, an effect attributed to HA’s bioactivity, while also potentially increasing mechanical strength when processed under conditions such as hot-pressing, which enhances interlayer polymer adhesion and homogenizes HA distribution.^18–22^ Materials such as poly­(lactic acid) (PLA), a biodegradable and biocompatible polyester, have been successfully obtained as fibers via SBS.^8,22^ Similarly, polycaprolactone (PCL), another biocompatible polymer with comparable biodegradability but distinct thermal properties and lower cost, has also been processed using this technique. Both PLA and PCL hold significant promise for biocomposite applications, particularly in tissue engineering, sustainable packaging and drug delivery systems.^23,24^ Differences in their thermal properties allow PCL to be melted or softened, after fiber production, forming a matrix to embed PLA fibers, similar to what is found in the structure of the *Rothschildia* sp. cocoons.^25–27^

In this sense, this work aimed to mimic the structure of the *Rothschildia* sp. cocoons to create bioinspired fibrous structures. In the first part, cocoons were characterized by scanning electron microscopy (SEM), differential scanning calorimetry (DSC), thermogravimetry (TG), and Fourier-transform infrared spectroscopy (FTIR) to understand their structure and morphology. In the second part, biocomposites with thermal and mechanical properties compatible with those found in the cocoons were produced by solution blow spinning PLA/PCL fibrous mats that underwent further heat treatment to melt PCL fibers, used as a matrix to encompass PLA fibers, and HA particles, used as a reinforcing agent, in order to obtain a biomimetic composite material.

## 2 Methodology

### 2.1 Materials

Cocoons of *R. speculifer* (*Rothschildia* sp.) moths were handpicked in Nova Palmeira/PB, located in the Northeast region in Brazil. PCL (Perstorp Capa 6500; Perstorp Química do Brasil Ltd. a–São Paulo/SP, Brazil; *M*_n_ = 50K g/mol, *T*_g_ = −60 °C, *T*_m_ = 58–60 °C) and poly­(l,d-lactic acid) (PLDA) (NatureWorks Ingeo 4060D; Cargill, São Paulo/SP, Brazil; *M*_n_ = 120 K g/mol, *T*_g_ = 55–60 °C, *T*_m_ = 155–160 °C), and hydroxyapatite, HA (LB Laborclin, Pinhais/PR, Brazil; ≥98% purity, Ca/P = 1.67) were used for fiber spinning, and chloroform (Labsynth, Diadema/SP, Brazil; P.A.—A.C.S., ≥99.8%) and acetone (Vetec Química, Duque de Caxias/RJ, Brazil; P.A. grade, ≥99.5%) were used as solvents.

### 2.2 Methods

#### 2.2.1 Characterization of the Cocoons

To understand properties and the structure of the cocoons, in order to mimic them, the following characterizations were performed:

##### 2.2.1.1 Scanning Electron Microscopy

Specimens were cut and analyzed on a LEO 1430 microscope (Carl Zeiss, Germany), performed using a secondary electron detector (SE detector), with acceleration voltage of 15 kV, a probe current of 60 pA, and an aperture size of 30.00 μm. Gold sputtering was performed using an Emitech K550X sputtering coating machine, promoting 40 nm coating thickness. Energy dispersive X-ray spectroscopy (EDS) was also used to confirm the presence of hydroxyapatite in the cocoons, and was performed concurrently with SEM imaging using identical sample preparation protocols. Subsequent image analysis was conducted using ImageJ version 1.54g (National Institutes of Health, USA; public domain) to quantify both fiber diameter and hydroxyapatite particle size distributions. For statistical reliability, all reported mean values, and standard deviations, were derived from 100 random measurements taken across multiple image fields for each sample. This systematic approach ensured representative characterization of the composite morphology.

##### 2.2.1.2 Thermogravimetry

Samples with a nominal weight of 8 mg were analyzed using a DTG-60H instrument (Shimadzu, Japan) in a platinum crucible under an argon atmosphere, with a flow rate of 50 mL/min and a heating rate of 10 °C/min, from room temperature to 1000 °C.

##### 2.2.1.3 Differential Scanning Calorimetry

DSC analyses were performed on samples with a nominal weight of 3 mg, placed in hermetic aluminum crucibles, using a DSC-60 Plus instrument (Shimadzu, Japan) under a nitrogen atmosphere with a flow rate of 50 mL/min. Solution blow spun samples underwent two heating cycles: the first cycle involved heating from room temperature (25–30 °C) to 250 °C at 40 °C/min, followed by cooling to 40 °C at 10 °C/min, and finally, a second heating up to 250 °C at 10 °C/min. The first heating cycle was carried out to emulate the fast heating of the fibers when placed in an oven at 90 °C.

##### 2.2.1.4 Fourier Transform Infrared Spectroscopy

KBr (potassium bromide) pellets for FTIR analysis, with a nominal weight of 150 mg, were prepared using a KBr-to-sample ratio of 100:1. Cocoons and HA were previously macerated in an agate mortar. All analyses were performed using an IRAffinity-1 FTIR spectrophotometer (Shimadzu, Japan). Spectra were recorded in the range of 4000–400 cm^–1^, averaging 64 scans at a resolution of 4 cm^–1^.

##### 2.2.1.5 Tensile Tests

Tensile test specimens were rectangular, with nominal dimensions of 30 mm × 10 mm and a thickness of 0.4 mm. Tests were conducted using a 3365 Instron universal testing machine (Instron, USA) equipped with a 5 kN load cell, at a crosshead speed of 1.0 mm/min. Due to the limited length of the cocoons, specimens could not be prepared according to standard dimensions; therefore, the 30 mm × 10 mm format was adopted, including those from cocoons and compressed multilayered structures. Seven specimens were tested for each configuration.

#### 2.2.2 Polymer Solutions

Three types of solutions were prepared using a 3:1 volume ratio of chloroform to acetone^13,28^ and maintained under stirring at room temperature, as shown in Table 1. After the specified mixing period, HA-containing (PLA + HA) solutions were immediately used for SBS processing to prevent HA agglomeration, while HA-free solutions could be refrigerated (4–8 °C) for short-term storage when needed.

**Table 1:** Parameters Used for Solution Preparation

polymer	concentration (% w/v, g/mL)	stirring time (h)
PCL	18% PCL	4
PLA	10% PLA	4
PLA + hA	10% (9% PLA and 1% HA)	12

#### 2.2.3 Fiber Spinning Process

The solution blow spinning system used in this study is capable of spinning with three different solutions simultaneously. The spinning setup consists of a matrix with three internal nozzles, eccentrically positioned within an outer nozzle and supplied with compressed air.^29^ Each internal nozzle is individually fed by a syringe containing a distinct polymer solution, all operated at the same ejection rate using an infusion pump (Insight Equipamentos Ltd., Ribeirão Preto/SP, Brazil). A detailed view of the multinozzle matrix is shown in Figure 1.

**Figure 1:**
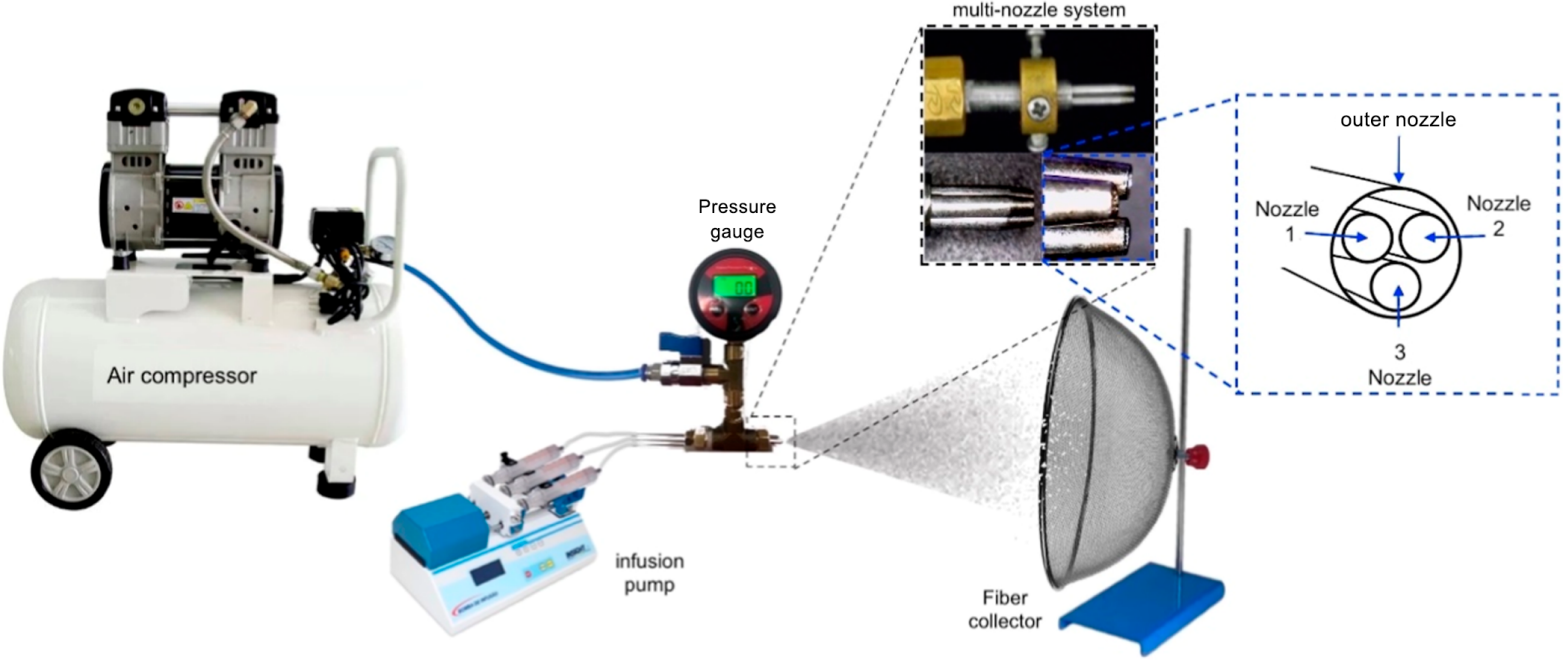
Schematic of the multinozzle matrix used for fiber spinning by SBS.

Five fiber combinations were produced, as described in Table 2. Pure PLA and PCL fibers were also spun to assess the feasibility of fiber production from these polymers for further characterization. The PCL/2PLA + HA and PCL/2PLA fibers were produced using the multinozzle setup, with nozzle 1 fed with 10 mL of PCL solution and the nozzles 2 and 3 fed with 10 mL each of the PLA + HA or PLA solutions. Prior to spinning, the PLA + HA solution was subjected to ultrasonic dispersion for 8 min at room temperature (25–30 °C) in an ultrasonic bath (Cristófoli Ltd., Campo Mourão/PR, Brazil) to ensure homogeneity. The amount of HA added corresponded to the content naturally found in the cocoons, as determined by thermogravimetric analysis.

**Table 2:** Specifications of the Produced Fiber Combinations Using the Multi-Nozzle System

polymer system	solution (nozzle)	type of nozzle	total volume (mL)
PCL	PCL (1)	single	10
PLA	PLA (1)	single	10
PLA + hA	PLA/HA (1)	single	10
PCL/2PLA + HA	PCL (1) + PLA/HA (2,3)	multi	30 (10 mL/nozzle)
PCL/2PLA	PCL (1) + PLA (2, 3)	multi	30 (10 mL/nozzle)

The same parameters were used for all fibers during the SBS production process, based on previous studies and preliminary tests conducted in the present study.^29^ Spinning process was carried out with an injection rate of 7.5 mL/h, a pressure of ∼138 kPa (20 psi), semihemispherical collector with a diameter of 25 cm and rotation speed of 180 rpm, a working distance of 25 cm, and a nozzle protrusion of 1.5 cm.

#### 2.2.4 Pressing of the Multilayered Structures

To replicate the morphology observed in cocoons, spun fiber mats were stacked and subsequently hot-pressed, with the number of layers adjusted as shown in Table 3. This number was determined through testing: samples with up to four layers were easily damaged, whereas those with seven or more layers showed poor visual consolidation.

**Table 3:** Description of the Procedures Used for Pressing Fiber Mats

group	fiber	number of layers
FIB1	PCL/2PLA + HA	6
FIB2	PCL/2PLA	6
FIB3	PCL/2PLA + HA	5
FIB4	PCL/2PLA	5

Given that PCL has a melting temperature of approximately 60 °C, a pressing temperature of 90 °C was selected to ensure PCL melting during the process while preserving the structural integrity of the PLA fibers, which have a higher melting temperature of around 150 °C. This approach aimed to embed PLA + HA fibers within a PCL matrix, thereby enhancing the mechanical strength of the resulting structure.^30^

The hot-pressing process was performed by applying a constant pressure of 1.925 kPa, calculated based on the mass of the metal plate (2.750 kg) distributed over an area of 0.014 m^2^ (Figure 2). The sample was then placed in the oven at 90 °C with the metal plate positioned atop to maintain the calculated pressure for 30 min. After this period, the sample and metal plate were removed together and allowed to cool at room temperature for an additional 30 min, ensuring safe demolding without structural damage to the sample. This controlled cooling protocol prevented thermal shock and preserved the composite’s integrity.

**Figure 2:**
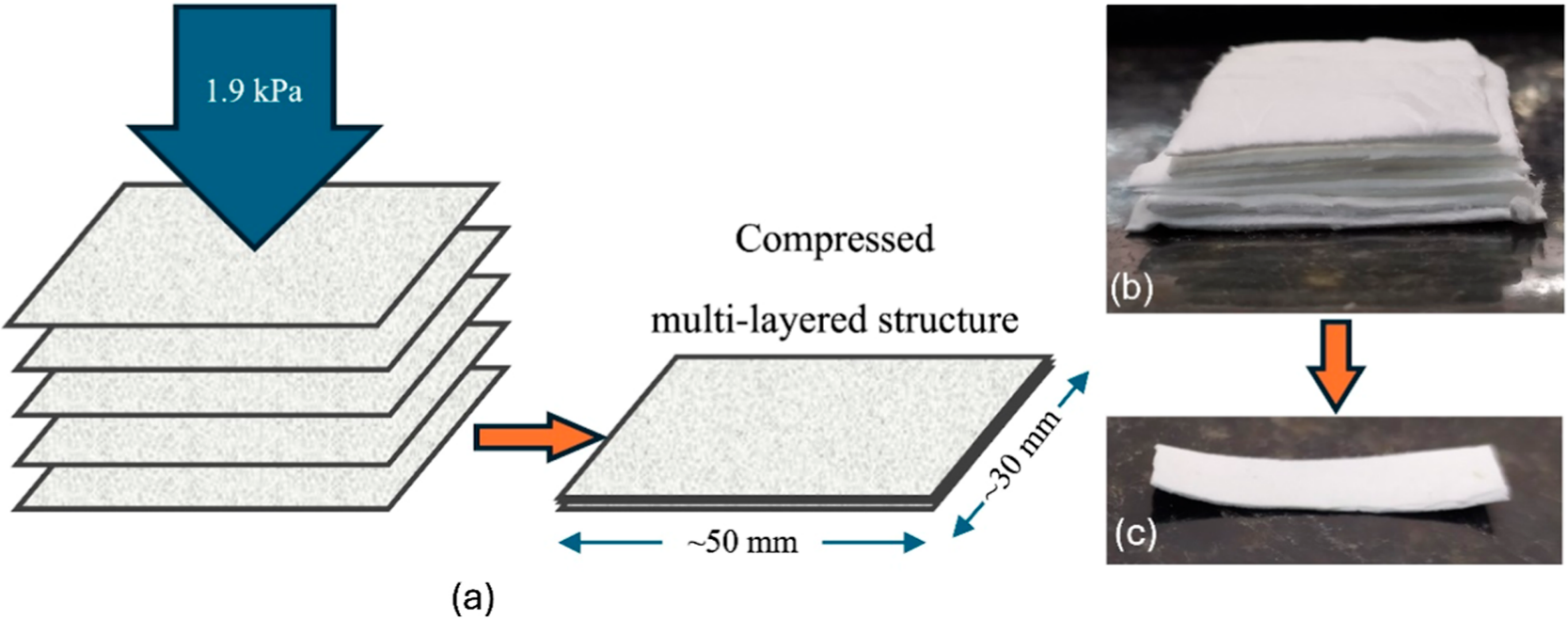
(a) Schematic of fiber mats stacked to form compressed multilayered structures; and images of these structures before (b) and after (c) pressing.

#### 2.2.5 Fiber Characterization

Fiber morphology was analyzed using a Hitachi TM3000 SEM (Chiyoda, Tokyo, Japan). Samples were gold-coated with an EMITECH K550X sputter coater and imaged at 15 kV accelerating voltage with magnifications ranging from 50× to 1500×.

Thermogravimetric (TG), differential scanning calorimetry (DSC), and Fourier-transform infrared spectroscopy (FTIR) analyses, as well as mechanical tests, were conducted using the same equipment and parameters as those for the cocoon samples. Approximately 3 mg of spun fibers cut from mats were used for the TG analysis. Mechanical tests were performed on samples cut from the four formulations (FIB1, FIB2, FIB3, FIB4), as shown in Figure 3.

**Figure 3:**
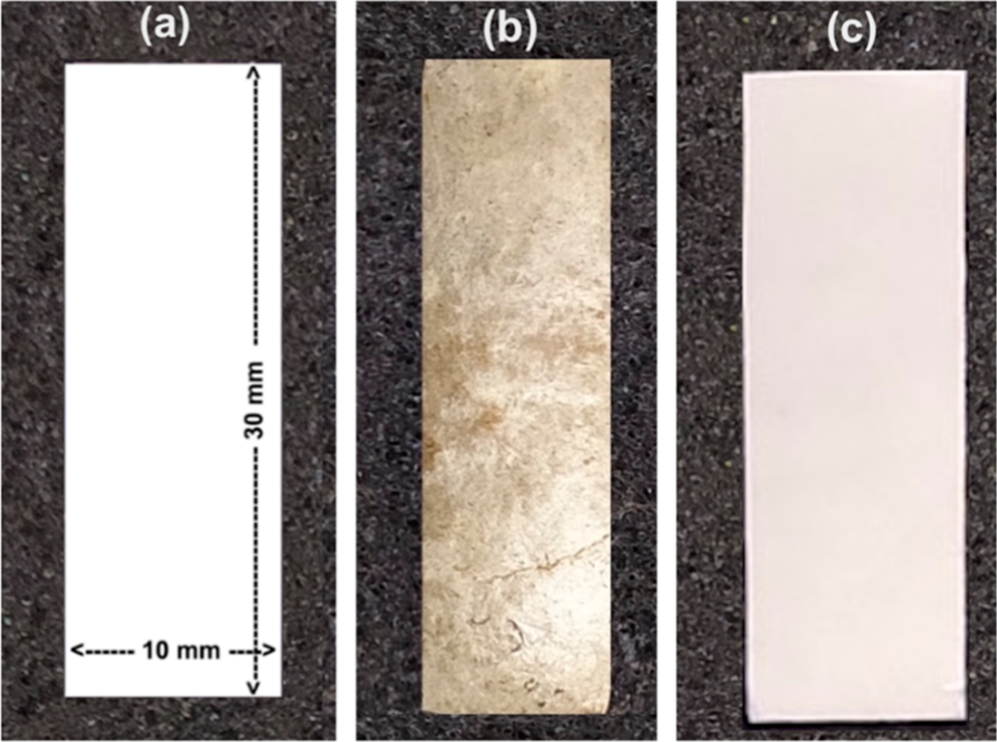
Specimens used in tensile tests: (a) schematic illustrating of sample dimensions and photographs of (b) cocoon, and (c) spun multilayer fibrous structures.

## 3 Results and Discussion

### 3.1 Cocoon Characterization

The cocoon exhibits a brownish coloration, an oval shape, and an approximate length of 6 cm. A Cocoons are visually distinct when the outer and inner surfaces are observed. SEM analysis of both sides (Figure 4) reveals randomly stacked ribbon-like structures composed of thicker fibers formed by bundles of thinner fibers. These finer fibers primarily consist of fibroin and are bound together by sericin, a protein that acts as a natural binder and can account for up to 25% of the cocoon’s composition.^5^ In cocoons of the *Rothschildia* sp., fibroin constitutes up to 70% of the total composition.^31^ The ribbon structures displayed an average width of 34 ± 13 μm, while the individual constituent fibers exhibited an average diameter of 1.06 ± 0.35 μm.

**Figure 4:**
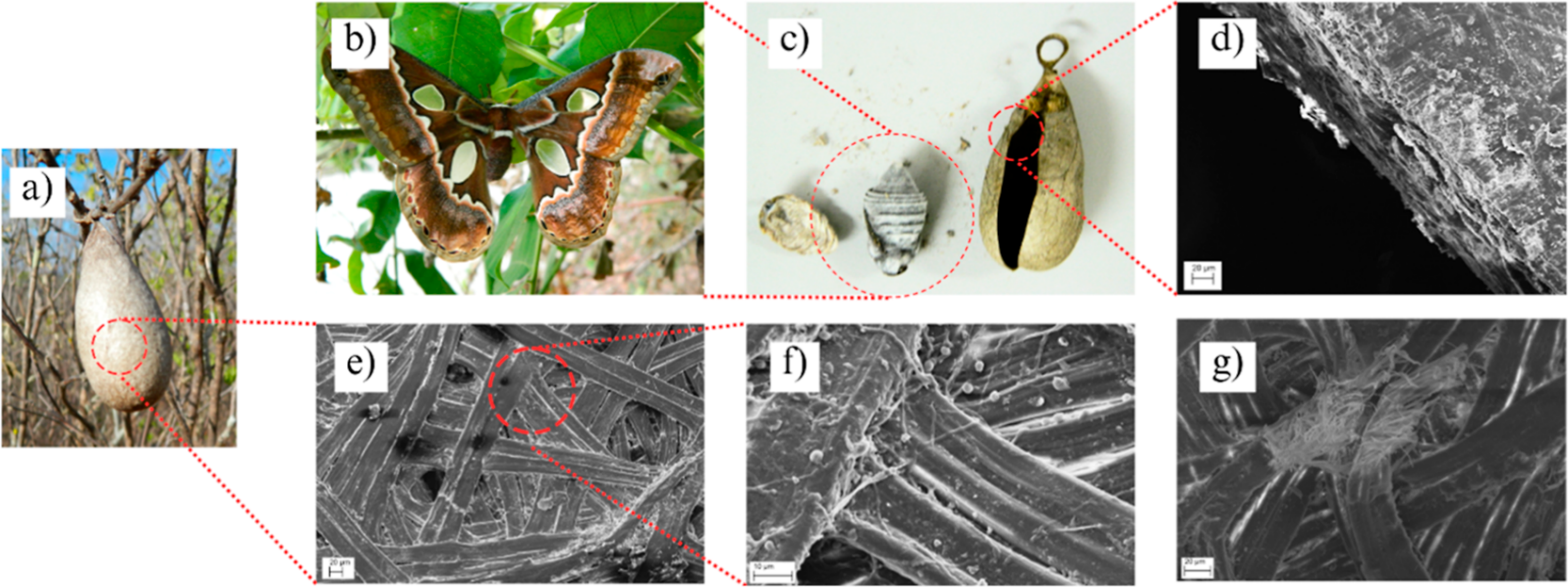
Images of *Rothschildia* specimens in their natural habitat (a,b). Illustration representing the regions analyzed via SEM (c). A multilayer structure made of ribbon-like structures which in turn are comprised by smaller fibers and hydroxyapatite particles, all bound together to form a flexible and strong cocoon (d-g).

The internal morphology of the cocoons differs markedly from the external surface, displaying particles with flat and well-defined faces characteristic of crystalline materials. To identify the composition of these particles, EDS analyses were conducted on both particles and fibers (Figure 5). Results revealed that calcium (Ca) is the primary component, confirming the presence of hydroxyapatite in cocoon’s composition, with particle sizes measuring 1.42 ± 0.47 μm. Magnesium (Mg), another constituent of hydroxyapatite, indicate partial Ca-substitution, a common feature in biological apatites, was also detected.^32–34^ In Figure 5a, the presence of ribbon-like structures is clearly observed, with an average width of 34.26 ± 13.17 μm and EDS peaks corresponding to chlorine (Cl) and potassium (K) were observed—elements commonly found in proteins.^35^ The presence of gold (Au) is attributed to the sputter-coating process used during sample preparation. The FTIR spectrum of the cocoons (Figure 5c) displays characteristic absorption bands of fibroin, with peaks at 1650 cm^–1^, 1500 cm^–1^, and 1230 cm^–1^ corresponding to amide I (C=O), amide II (N–H), and amide III (C–N), respectively.^31,36^ Additionally, a band around 3250 cm^–1^indicates the presence of O–H groups, consistent with previous reports.^37^

**Figure 5:**
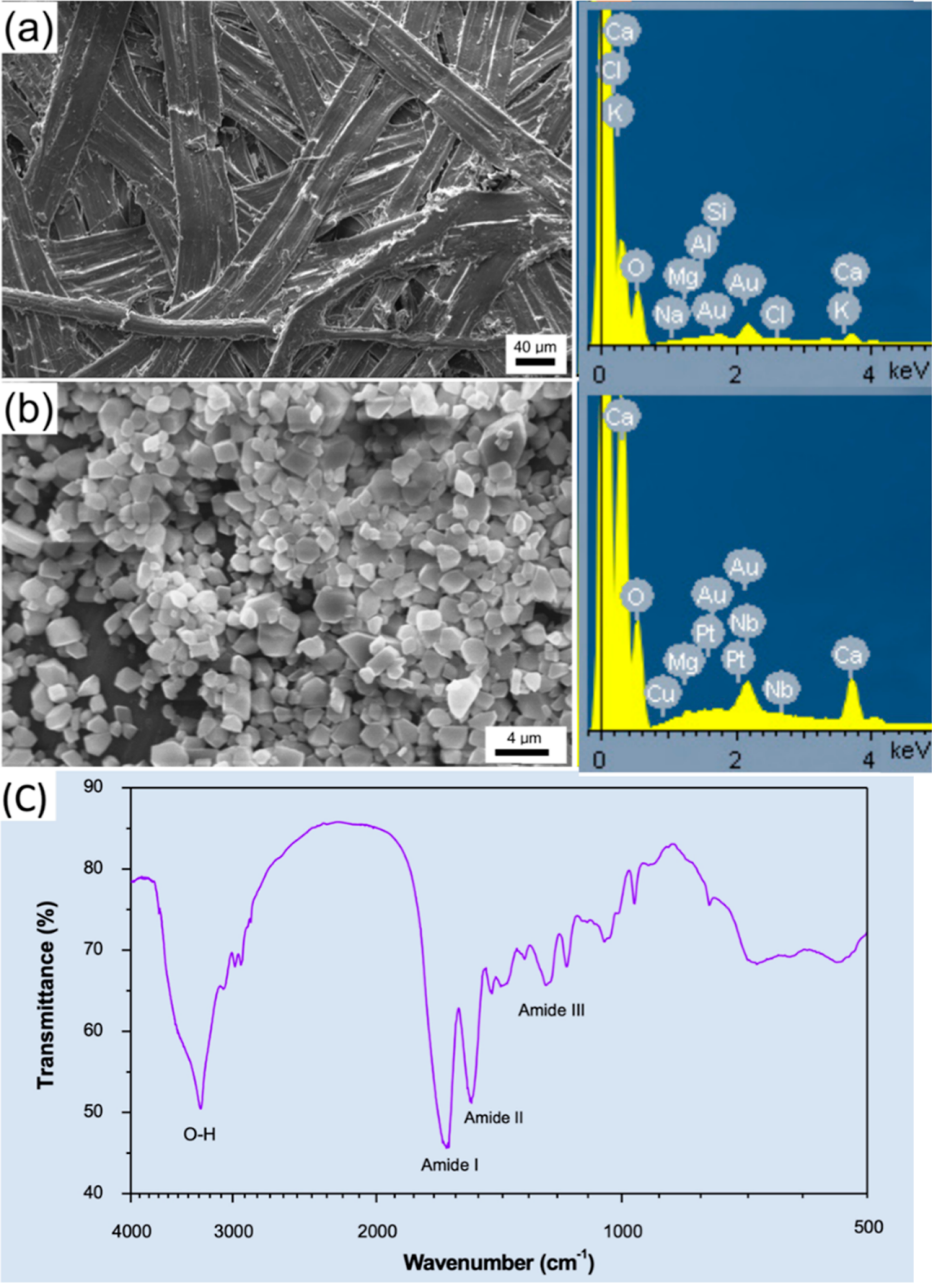
SEM images of the cocoons showing (a) the ribbon-like structures (outside) and (b) hydroxyapatite particles (inside) sided by EDS results (on the right); (c) FTIR spectrum obtained of the cocoons.

The thermal properties of the cocoons were accessed using TG and DSC, as shown in Figure 6. Thermogravimetric data in Figure 6a reveal three distinct mass loss events: an initial mass loss beginning at 60 °C (attributed to evaporation of physically adsorbed water); a secondary loss at 140 °C (corresponding to bound water release from hydrophilic protein domains); followed by the main degradation event initiating at 290 °C due to thermal decomposition of fibroin peptide chains, with complete degradation occurring by 975 °C.^38^ DSC results (Figure 6b) show an endothermic peak at 70 °C, corresponding to the onset of water evaporation. At 120 °C, a baseline shift is observed, which is not characteristic of fibroin itself, but likely due to the presence of HA.^39^

**Figure 6:**
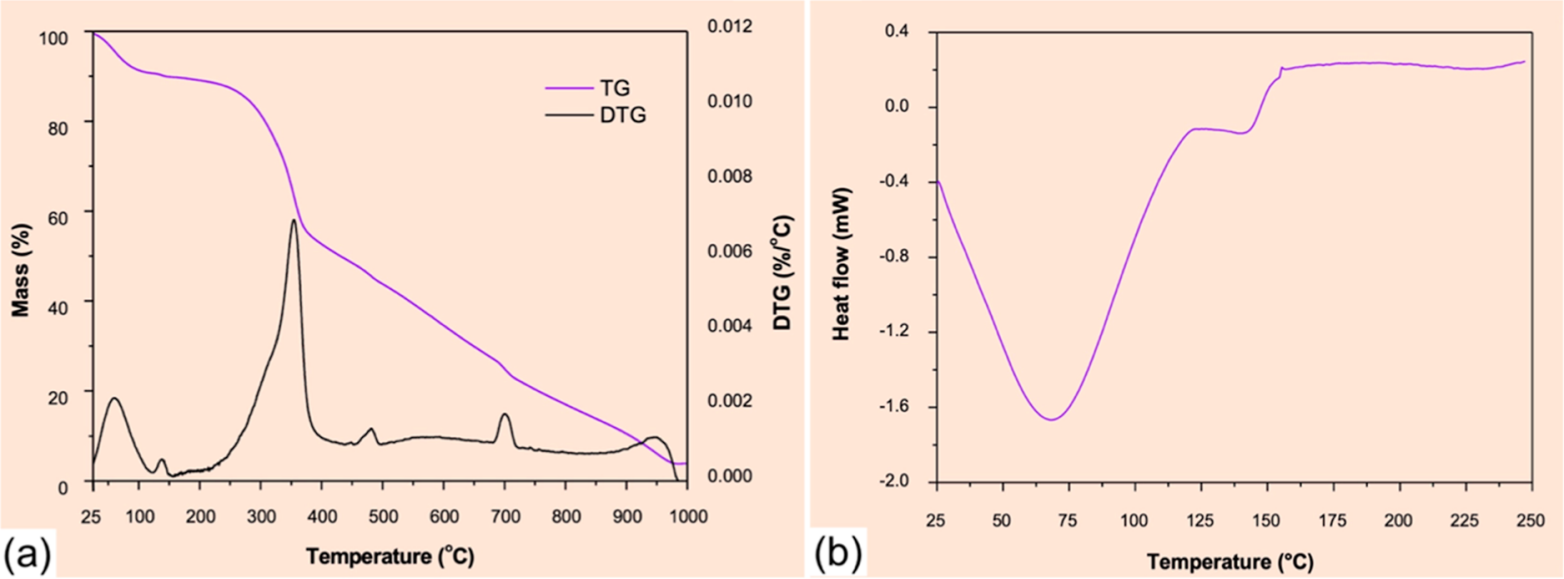
TG (a) and DSC (b) curves of the cocoons.

### 3.2 Characterization of Solution Blow-Spun Fibers

To mimic the structure of the cocoons, as described in the experimental section, a multinozzle SBS apparatus (Figure 1) was employed to spin individual PCL and PLA filaments from the polymer solutions outlined in Tables 1 and 2. These filaments intermesh to form nonwoven fibrous mats, which were then subjected to a heat-treatment process to melt the PCL while preserving the PLA fibers. The resulting PCL matrix mimics the sericin matrix surrounding the fibroin bundles in natural cocoons. Furthermore, the compression of several fiber layers, each consisting of intermeshed fibers, partially replicates the multilayered structure of the cocoons spun by the *Rothschildia* sp. moths. These cocoons are typically suspended between leaves and firmly attached to branches of marmeleiro (*Croton blanchetianus*) and velame (*Croton heliotropiifolius*) trees in the Northeastern Brazilian semiarid region.^40^

SEM images of the PCL/2PLA (Figure 7a) and PCL/2PLA + HA (Figure 7b) mats prior to heat-treatment/pressing process reveal a random dispersion of PCL and PLA fibers. The average fiber diameter was 1.44 ± 0.49 μm for PCL/2PLA 0.87 ± 0.48 μm for PCL/2PLA + HA, showing a reduction in fiber diameter in samples containing HA. Following heat-treatment and compressing the spun mats and mat stacks (FIB1, FIB2, FIB3, and FIB4), the morphology observed in Figure 7c shows a molten PCL matrix that binds the PLA fibers. SEM images at increasing magnifications highlight the resulting porous microstructure in greater details.

**Figure 7:**
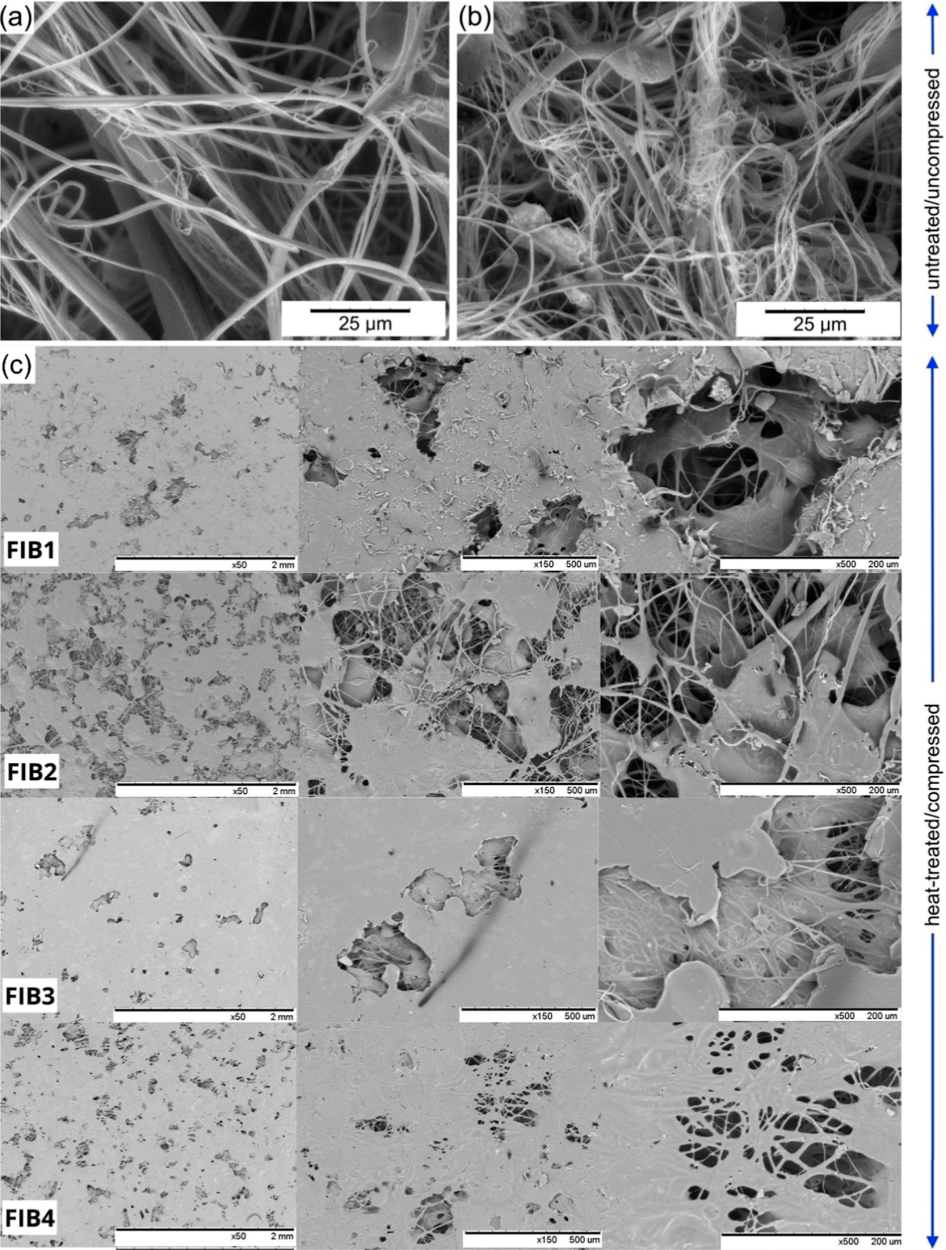
SEM images of PCL/2PLA solution blow-spun fibers without (a) and with hydroxyapatite (b) before heat-treatment/pressing; and (c) and after heat-treatment and pressing.

The morphology of the spun fibrous mats bears some resemblance to that of natural cocoons, with the key distinction being fiber arrangement. While cocoons exhibit a highly organized fiber structure, the PLA fibers in the PCL matrix are randomly dispersed. This difference is primarily attributed to the use of a semihemispherical collector in the SBS process. Aligned fibers can be achieved using cylindrical collectors, operating at high speeds, as reported in previous studies.^41,42^ Furthermore, the relative content of PCL and PLA in the SBS fibers can be tuned to tailor both structure and properties. As shown in Table 3, this can be achieved by varying the nozzle configurations, polymer ejection rates, and polymer concentration across the three nozzles. An increase in PCL content enhances the mechanical properties of the spun multilayered structures by reducing pore size. Conversely, a more porous structure can be more beneficial to certain applications such as in tissue engineering, where larger surface areas promote cell adhesion and proliferation.^43–45^ In addition, the hydroxyapatite content within the fibers can be adjusted to support bone regeneration, given its crucial role in osteogenesis.^46,47^

TG analysis (Figure 8a) indicates that PCL begins to degrade at approximately 284 °C, with 95% mass loss occurring at 432 °C. PLA exhibits a similar thermal degradation profile, with decomposition occurring around 290 to 385 °C. DSC analysis (Figure 8b) shows a slight baseline shift at 38 °C for PCL, corresponding to its glass transition temperature (*T*_g_), followed by a distinct endothermic melting peak at 63 °C. For PLA, a baseline shift at 47 °C indicates its *T*_g_, along with an exothermic crystallization peak at 114 °C and an endothermic melting peak at 142 °C. These thermal transitions guided the selection of an optimal heat-treatment temperature - to melt PCL phase while preserving the structural integrity of the PLA fibers, which have a significantly higher melting temperature. This selective melting behavior is further corroborated by SEM analyses, which reveal PLA fibers within a continuous PCL matrix. Moreover, both TG and DSC analyses confirm that the thermal behavior of the PCL/2PLA and PCL/2PLA + HA closely resembles that of a physical mixture of the pure polymers.^48,49^

**Figure 8:**
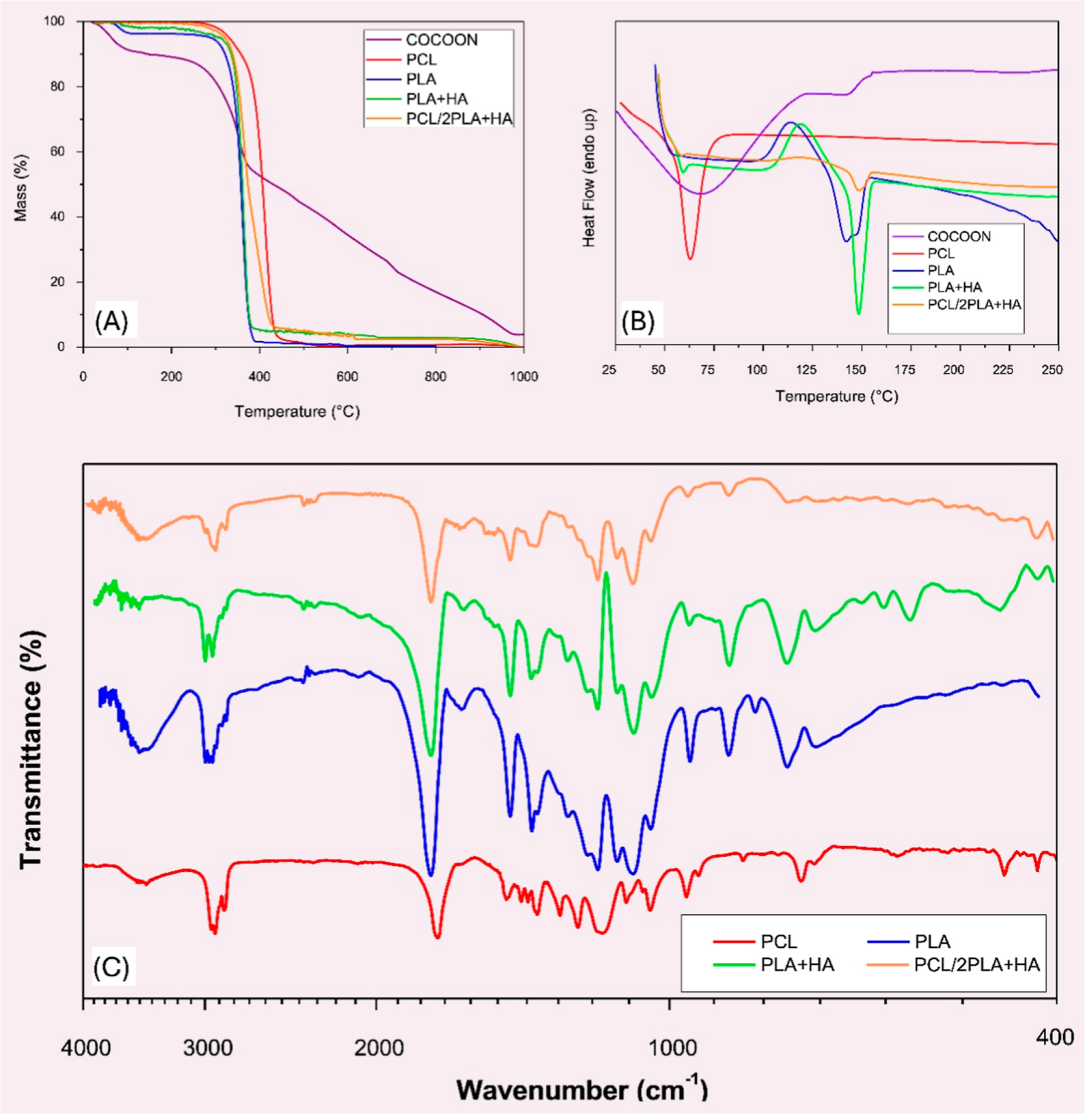
Thermal and FTIR analyses of cocoon and spun fibers: (a) TG curves; (b) DSC curves and (c) FTIR spectra.

Fiber processing by SBS did not induce any chemical composition alterations, in agreement with previous reports^50^ and supported by the FTIR spectra of the spun fibers (Figure 8c). The FTIR spectrum of PCL displays an absorption band at 1750 cm^–1^ corresponding to C=O stretching, two bands around 3000 cm^–1^ associated with C–H stretching, and additional peaks between 1500 and 1000 cm^–1^ attributed to C–O stretching and vibrational modes. For PLA, a band at 3507 cm^–1^ is observed due to O–H stretching, along with bands around 3000 cm^–1^ (C–H stretching), a peak at 1750 cm^–1^ (C=O stretching), and a distinct band at 1190 cm^–1^ related to C–O bonding.^51^ The spectra of the PLA + HA and PCL/2PLA + HA samples show minimal variation compared to the pure polymers. The primary difference arises from characteristic hydroxyapatite (HA) peaks, notably those between 3600 and 3400 cm^–1^ due to the O–H stretching, and bands near 600 cm^–1^ and 1000 cm^–1^ corresponding PO_4_^3–^ groups.^52–54^

Tensile tests were carried out on both cocoon and heat-treated spun fiber mats, as shown in Figure 9. Seven cocoon samples were initially tested, yielding an average maximum load of 162.8 ± 25.1 N and an average tensile strength of 38.0 ± 7.2 MPa (Figure 9a). The stress–strain curves exhibited typical ductile behavior, characterized by plastic deformation prior to failure, but without evidence of chain slippage, as previously reported.^55^ The solution-blow spun fibers (Figure 9b) exhibited lower tensile strength and ductility compared to the cocoons, as expected. Nonetheless, the mechanical performance—both in terms of average stress and overall stress–strain behavior—was considered adequate for prospective applications in bone regeneration, particularly in nonload-bearing defects.^56^

**Figure 9:**
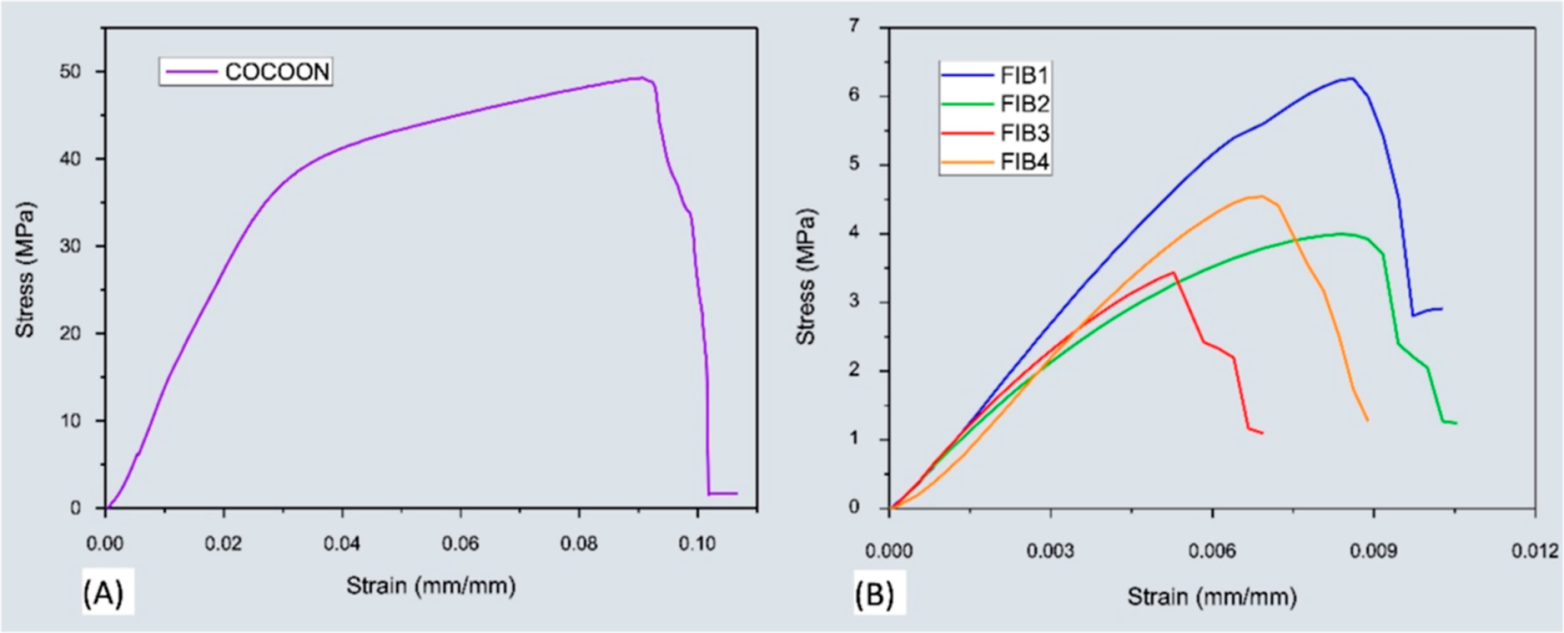
Representative stress–strain curves of: (a) cocoons and (b) fiber mats after heat treatment.

The average mechanical properties of the four spun fiber samples—prepared under different conditions as outlined in Table 3—are summarized in Table 4. These results were used to compare the mechanical performance of the heat-treated mats with that of the natural cocoons. As shown in Figure 9 and Table 4, the number of stacked layers played a key role in determining the mechanical strength of the samples.^57^ Among them, the FIB1 and FIB2 samples, both pressed with six layers exhibited superior mechanical performance, with FIB1 achieving the highest strength.

**Table 4:** Tensile Properties for the Cocoons of *Rothschildia* sp. and PCL/PLA Multi-Layered Fibrous Structures Produced by Solution Blow Spinning

material	maximum load (N)	maximum stress (MPa)
cocoon	162.8 ± 25.1	38.0 ± 7.2
FIB1	50.7 ± 12.4	6.0 ± 1.7
FIB2	52.1 ± 16.8	4.1 ± 1.1
FIB3	20.5 ± 4.4	3.2 ± 0.8
FIB4	28.7 ± 5.4	5.0 ± 1.7

Analysis of the influence of hydroxyapatite (HA) on mechanical performance revealed a reduction in average maximum load for HA-containing samples. Specifically, samples without HA (FIB2 and FIB4) exhibited higher maximum load and stress values compared to those containing HA (FIB1 and FIB3). As observed by Wojasiński et al. (2024), hydroxyapatite (HA) typically enhances the compressive strength of polymer composites.^58^ However, in our tensile testing, the presence of HA reduced the maximum load. This trend was consistent across both the six-layer and five-layer groups. The presence of HA appears to introduce localized defects within the fiber structure, consistent with the previously observed decrease in the diameter of HA-loaded PLA fibers. Conversely, increasing the number of layers led to more compact morphology, characterized by a greater proportion of molten PCL matrix surrounding PLA fibers. This enhanced mechanical strength of the spun and heat-treated multilayered structures. In summary, an optimized combination of PCL/PLA ratios, number of stacked layers, HA incorporation - followed by thermal consolidation under compression—enables the fabrication of porous, multilayered mats via solution blow spinning. These structures that closely mimic the natural architecture of *Rothschildia* sp. cocoons hold promise for a wide range of applications, including biocomposites and scaffolds for tissue regeneration.

## 4 Conclusions

Analysis of the *Rothschildia* cocoon revealed an oval-shaped structure, approximately 6 cm in length, and consist of randomly stacked, ribbon-like thick fibers (average width of 34 ± 13 μm), composed of bundled thinner fibers (average diameter of 1.06 ± 0.35 μm), primarily made of fibroin and magnesium-doped hydroxyapatite bound by sericin. To mimic this natural architecture, we engineered fibrous mats via solution blow spinning of PLA and PCL with and without hydroxyapatite. Four distinct fibrous mats were produced by varying polymer components and layer numbers, which underwent hot-pressing to yield morphologies and mechanical characteristics closely resembling those of the cocoon. The FIB1 sample (6 layers of PCL/2PLA + HA) exhibited the highest tensile strength (6.0 ± 1.7 MPa), while thermal analyses confirmed stability up to 280 °C. Crucially, the hot-pressing process at 90 °C enabled selective PCL melting while preserving PLA fiber integrity, creating a hierarchical structure that replicates the cocoon’s mechanical synergy. These results demonstrate not just morphological but functional biomimicry, with specific application potential in nonload-bearing bone regeneration. This work establishes a reproducible platform for designing bioinspired composites with tailored properties, with immediate translation potential in orthopedic scaffolds.
